# Effectiveness of gamified digital interventions in mental health prevention and health promotion among adults: a scoping review

**DOI:** 10.1186/s12889-023-17517-3

**Published:** 2024-01-02

**Authors:** Leona Aschentrup, Pia Anna Steimer, Kevin Dadaczynski, Timothy Mc Call, Florian Fischer, Kamil J. Wrona

**Affiliations:** 1 School of Public Health, Bielefeld University, Bielefeld, Germany; 2 Bielefeld University of Applied Sciences and Arts, Bielefeld, Germany; 3 Department of Global Public Health, Karolinska Institute, Solna, Sweden; 4 Department of Health Sciences, Fulda University of Applied Sciences, Fulda, Germany; 5 Centre for Applied Health Sciences, Leuphana University Lueneburg, Lueneburg, Germany; 6 Medical School OWL, Bielefeld University, Bielefeld, Germany; 7 Institute of Public Health, Charité – Universitätsmedizin Berlin, Berlin, Germany; 8 Bavarian Research Center for Digital Health and Social Care, Kempten University of Applied Sciences, Kempten, Germany

**Keywords:** Gamification, Games for health, Digitalization, Mental health, Prevention, Health promotion

## Abstract

**Background:**

Though still a young field of research, gamified digital interventions have demonstrated potential in exerting a favourable impact on health and overall well-being. With the increasing use of the internet and digital devices, the integration of game elements presents novel opportunities for preventing mental disorders and enhancing mental health. Hence, this review aims to assess the effectiveness of gamified interventions focusing on preventing mental disorders or promoting mental health among adults.

**Methods:**

Based on a scoping review across four databases (MEDLINE, Embase, PsycInfo and Web of Science), 7,953 studies were initially identified. After removing duplicates and screening titles, abstracts and full texts, 16 studies were identified as suitable for inclusion in a narrative synthesis of findings. We included interventional studies encompassing an intervention and a control group aiming to investigate the effectiveness of the use of gamified digital mental health interventions and the use of gamified digital elements.

**Results:**

Overall, positive effects of gamified interventions on mental health-related outcomes were identified. In particular, beneficial consequences for psychological well-being and depressive symptoms were observed in all studies. However, further outcomes, such as resilience, anxiety, stress or satisfaction with life, showed heterogenous findings. Most game elements used were reward, sensation and progress, whilst the quantity of elements was not consistent and, therefore, no substantiated conclusion regarding the (optimal) quantity or composition of game elements can be drawn. Further, the outcomes, measurements and analyses differed greatly between the 16 included studies making comparisons difficult.

**Conclusion:**

In summary, this review demonstrates the potential of integrating digital game elements on mental health and well-being with still a great gap of research. A taxonomy is needed to adequately address relevant game elements in the field of mental health promotion and prevention of mental disorders. Therefore, future studies should explicitly focus on the mechanisms of effect and apply rigorous study designs.

**Supplementary Information:**

The online version contains supplementary material available at 10.1186/s12889-023-17517-3.

## Background

The mental health of individuals is affected by a variety of determinants on different levels such as individual or social factors, economics and culture, as well as living and working conditions, environmental and biological factors [1]. In 2016, about 16% of the global population was affected by mental or addictive disorders [2]. The most common mental disorders are depression (prevalence per 100,000: 3,627) and anxiety disorders (prevalence per 100,000: 3,715) [2]. In 1990, the global prevalence of depression and anxiety disorders was about 12.7%, proving them to be the most common mental disorders for at least 30 years [3]. Moreover, within the COVID-19 pandemic the prevalence has considerably increased in most countries [4]. Nochaiwong et al. [5] have also indicated the impact of the COVID-19 pandemic on the global prevalence of mental health problems among the general population.

For counteracting these challenges, digital technologies may be supportive. At the moment, the whole health sector is undergoing a monumental shift: Digital technologies are shaping the present and the future of health care. Additionally, the interest in online health information continues to grow. Along with the growing use and relevance of the Internet worldwide (2005: 1 billion; 2022: 5.3 billion) [6], the increasing relevance of digital applications and media results in new possibilities and potentials for health promotion and prevention, especially in terms of mobile and web-based applications [7]. Moreover, the digital transformation reveals changes on a social, organisational and individual level [8]. For targeting changes on those levels, innovative digital methods and concepts such as gamification have been utilized increasingly. Gamification is defined as the utilization of game-design techniques and elements outside of a game-context to positively impact user behaviour [9, 10]. The game elements cannot always be clearly separated, but largely refer to typical characteristics of a game [9]. In contrast to serious games – which we do no focus upon in this contribution –, it is not about the complete game, but about playful elements. Positive effects on health and well-being through gamification have been observed [11–15]. Thus, e.g., Johnson et al. [11] identified within their systematic review that the majority of studies found positive effects on health and well-being through gamified elements. In addition, Bostock and colleagues [15] found within their randomized controlled trial a significant positive association with gamification and well-being, likewise with stress. Further, in an multi-centre interventional study, gamification has been proven to be an effective strategy for prevention of diseases and helps reducing expenses in prevention [16]. Thus, gamified interventions can induce behaviour change by improving self-determination and self-management skills [17]. In addition, continuous use of such applications increases also the satisfaction and self-esteem [18]. Overall, gamification seems to be an effective strategy to promote health. Previous reviews already investigated gamified interventions and the effect on mental health [11–14]. However, current literature has not comprehensively focused on prevention and health promotion [11–13]. Previous research such as by Six et al. [13] examined the effectiveness of gamification in mental health apps to reduce depression symptoms of adults, regardless of whether they are sick or not. Cheng et al. [14] in turn analysed which game elements and mental health and well-being domains are most commonly utilized and targeted in interventions of gamification for mental health and well-being. In this respect, further research is required in terms of whether and how gamified digital interventions can promote mental health and prevent mental disorders among adults.

In this context, we consider two research questions to be relevant: How effective is the use of gamified interventions, measured by relevant indicators for improving mental health or preventing mental diseases, for working-age adults? And secondly: Which game elements are most commonly used within the interventions identified?

## Methods

Originally planned as a systematic literature review, we performed a screening in four major databases, namely MEDLINE (via PubMed), Embase, PsycInfo and Web of Science. The primary objective was to identify intervention studies featuring at least one control condition that have been published between 2010 and 2022. The search was executed in January 2023. To ensure consistency and comparability, we used the following complete search algorithm, based on similar reviews [11, 14]:

- “mental health” and “well-being” were included as terms with positive connotations since well-being was seen as a mental health-related outcome in this study;
- “mental disorders” and “mental illness” as terms with negative connotations;
- and “depression” as well as “anxiety” as the most common disorders in this context. These two indications were chosen due to their high prevalence and importance in the field of mental disorders as already described in the background section, but also other indications were included if identified by the search algorithm.

Thus, the following search strategy was utilised:

(gamif* OR game* OR playful*) AND (mental health OR wellbeing OR (mental illness* AND prevent*) OR (mental disorder* AND prevent*) OR (depress* AND prevent*) OR (anxi*AND prevent*))

**Fig. 1:**
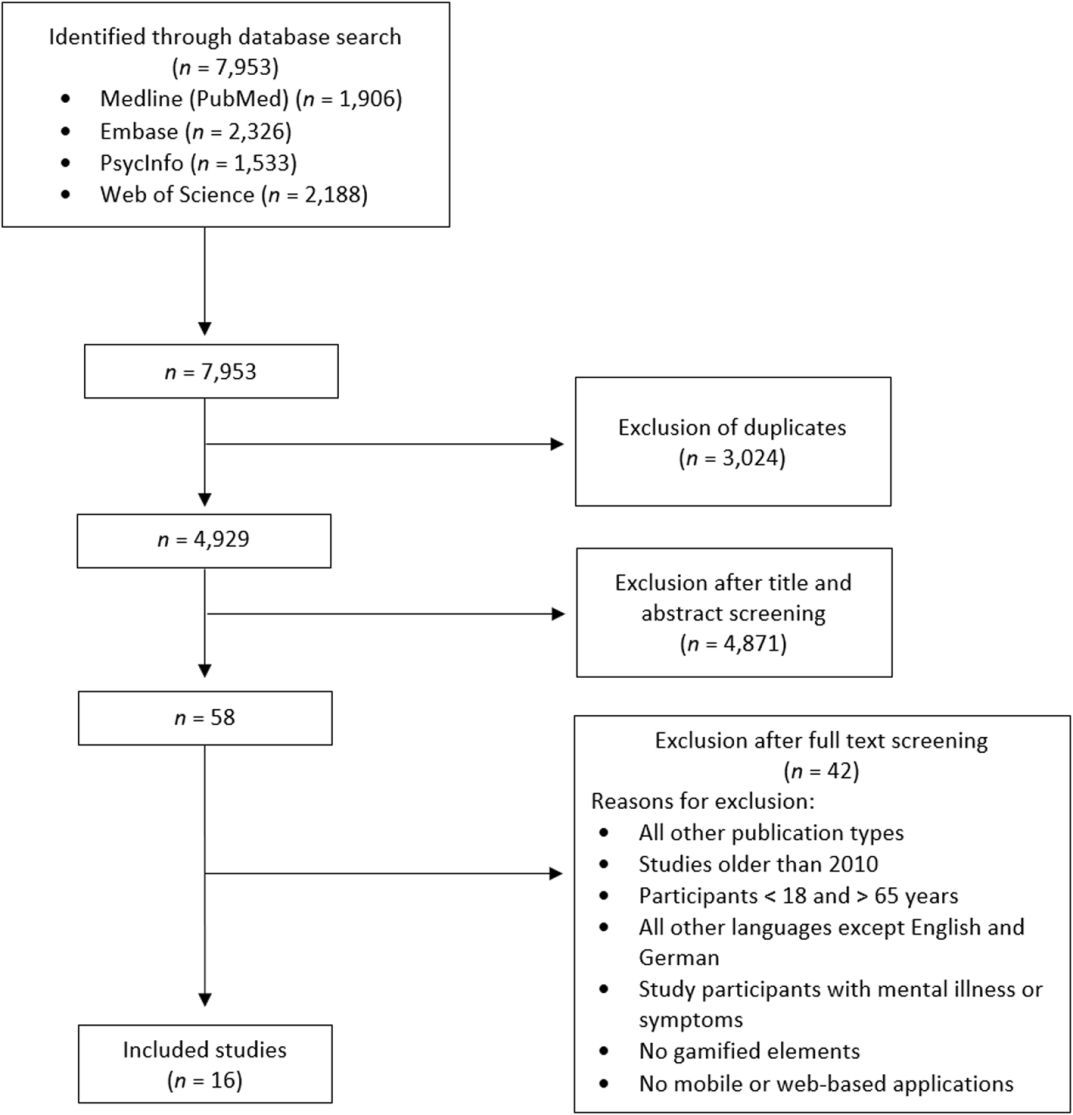
Screening process

The database search yielded a total of 7,953 records, of which 3,024 duplicates were removed. The study selection process started with the screening of titles and abstracts. Two authors (LA and PAS) independently carried out the initial title and abstract screening, which led to an interrater-agreement of 96.4%. In case of inconsistency a third party (FF or KW) screened those abstracts. The screening of titles and abstracts led to the exclusion of 4,871 studies. The screening of references of systematic reviews identified within the database search and studies included in the full text screening has not led to further hits. Subsequently, two authors (LA and PAS) independently appraised the full-texts against the inclusion and exclusion criteria (Table 1) and any discrepancies (*n* = 8) were resolved by consensus. Finally, the full text screening for the remaining 58 records resulted in 16 included studies (see Fig. 1).

**Table 1:** Inclusion and exclusion criteria

Inclusion criteria	Exclusion criteria
Study types: Interventional studies comparing an intervention and a control group	All other study types (e.g., studies without control group, qualitative studies)
Publication date: 2010–2022	Older than 2010
Age group of working age (18–65 years) included	Participants < 18 and > 65 years of age
Language: English and German	All other languages then English and German
Healthy study participants	Study participants with mental illness or symptoms
Game elements included	No game elements included
Mobile or web-based interventions only	No mobile or web-based interventions

Results are presented in accordance with the PRISMA statement [19]. Due to the high heterogeneity of studies, we were unable to compare effect estimates as originally planned. For that reason, we decided to shift the systematic literature review to a scoping review for synthesizing the results. We extracted information on the study design (e.g., sample size, drop-outs, number and time of follow-ups, primary and secondary outcomes as well as scales for measuring these outcomes) and intervention (e.g., country where the study was conducted, duration and characteristics of intervention and control group). For the latter, we additionally prepared a documentation of game mechanics based on the studies by Toda et al. [20] and Hervas et al. [21] to investigate the use of game elements in mental health promotion and prevention. Thus, the following game elements were included: Reward, Sensation, Progress, Challenges, Surprise, Storytelling/Narration, Social sharing, Level, Leaderboard, Goals, Avatar. For an explanation of the elements used in this study, see supplementary material. The results are described as a qualitative overview, allowing for a systematization of the outcomes and categorization of game elements.

An assessment of the methodological quality of the included randomized controlled studies was conducted by two authors (LA and FF) independently. The assessment was based on the revised Cochrane risk-of-bias tool for randomized studies (RoB2), which also allows to assess the quality of cluster-randomized studies [22]. No discrepancies in assessment were observed.

## Results

A total of 16 studies were identified and included in the synthesis on the effectiveness of gamified digital interventions on mental health prevention and promotion among adults (Table 2). The measurements, statistical analyses and outcomes differed greatly; however, all studies showed overall a positive impact on mental health outcomes in at least one related outcome measurement.

**Table 2:** Characteristics of included studies

Reference	Study design/ Number and time of follow-ups	Country	Sample^1^ and drop-outs	Game-based elements	Duration	Intervention
Bostock et al. (2019) [15]	RCT / 2 (after 8 weeks, after 9–11 weeks)	United Kingdom	*Age:* M = 35.5 (23–61 years) ***Intervention group (n* = *128):*** *Female sex:* 60.2% *Drop-out:* 18.0% ***Control group (n* = *110):*** *Female sex:* 58.2% *Drop-out:* 26.4% ***Handling of drop-outs:*** Per protocol analysis	- Reward - Progress - Surprise - Sensation^2^	8 weeks	Intervention group: - Participants had 8 weeks to use the app “HeadSpace” with 45 meditation sessions. One session should be completed each day. A weekly reminder was sent from the research team Wait-list group: - The control group was once sent a link to an online advice for work stress from the NHS
Champion et al. (2018) [23]	RCT / 2 (afterday 10, after day 30)	United Kingdom	***Intervention group (n* = *38):*** *Age:* M = 40.2 *Female sex:* 44.8% *Drop-out:* 23.7% ***Control group (n* = *36):*** *Age: M* = *38.2* *Female sex:* 72.7% *Drop-out:* 8.3% ***Handling of drop-outs:*** Intention-to-treat analysis + complete case analysis	- Reward - Progress - Surprise - Sensation^2^	30 days	Intervention group: - Participants had 30 days access to the self-guided mindfulness meditation app and were encouraged to use the app for 10–20 min daily. 3 levels with 10 sessions to be completed each. Further, e-mails were sent with questionnaires and notes to encourage them to continue Wait-list group: - Participants received only the questionnaires and follow-up emails informing them that they would have access to the app after 30 days
Collins et al. (2019) [24]	Experimental and field study with randomization/ 2 (after presentation of task/after break activity, after 10 min or 5 days past intervention)	United Kingdom	**Study 1 (*n* = 45)** *Age:* 19–36 years *Female sex: 57.8%* *Drop-out:* 8.9% (due to technical issue) **Study 2 (*n* = 20)** *Age:* 19–58 years *Female sex:* 60.0% *Drop-out:* No information ***Handling of drop-outs:*** No information on numbers of participants at intervention and control group Per-protocol analysis	- Reward - Progress - Surprise - Sensation^2^	**Study 1:** 10 min for the intervention **Study 2:** 5 days	**Study 1:** Intervention group (Mindfulness app): - Participants used a 10 min mindfulness exercise from HeadSpace during their 10 min break Control group: - Participants played Block! Hexa Puzzle during their 10 min break Control group: - Participants were just sitting in a room and resting, and could use the toy called fidget spinner during their 10 min break **Study 2:** The same tasks were for the digital games group and the mindfulness app group as in study 1, except that participants had to do their after-work break activity for 10 min every 5 days. A daily email reminder was sent. No other control group was included
Costa et al. (2018) [25]	RCT / 1	Portugal	***Intervention group (n* = *20):*** *Age:* M = 73 *Female sex:* 50.0% ***Comparison group (n* = *20):*** *Age:* M = 69 *Female sex:* 60.0% ***Control group (n* = *20):*** *Age:* M = 69 *Female sex:* 65.0% ***Handling of drop-outs:*** No drop-outs	- Challenges - Storytelling - Social sharing - Sensation	6 weeks	Intervention group: - Participants first tested the Game-Based Learning Program (GBLP) which includes a set of missions related to physical and cognitive activity by travelling to Hizen, 1709. Further, mini-games could be played. In the second part, they used a computer-assisted platform (CAP) with videos on cognitive activity, nutrition, or human security topics. At the end, users could share their progress and daily life missions Comparison group: - Same intervention as in the intervention group, just the other way around Wait-list group: - No intervention
Deady et al. (2022) [26]	RCT / 3(after 5 weeks, after 3 months, after 12 months)	Australia	***Intervention group (n* = *1,131):*** *Age:* M = 40.2 (18–78 years) *Female sex:* 27.6% *Drop-out:* 0.3% ***Control group (n* = *1,144):*** *Age:* M = 40.3 (18–68 years) *Female sex:* 24.0% *Drop-out:* 0.1% ***Handling of drop-outs:*** Intention-to-treat analysis	- Challenges	30 days	Intervention group: - Participants had 30 days of access to the behavioural activation and mindfulness-based app HeadGear. They needed to complete 5–10 min challenges per day Control group: - Participants used for 30 days an app similar to HeadGear, which includes a risk calculator and mood tracker
Economides et al. (2018) [27]	RCT / 1 (after 1–2 months)	-	*Age:* 18–49 years ***Intervention group (n* = *41):*** *Female sex:* 63.4% ***Control group (n* = *28):*** *Female sex:* 53.6% ***Handling of drop-outs:*** No detailed information on drop-outs stratified by intervention vs. control. Overall drop-out 27.5% Per-protocol analysis	- Reward - Progress - Surprise - Sensation^2^	1 month	Intervention group: - Participants had one month of access to the app HeadSpace and needed to complete the first 10 introductory sessions including an introduction to meditation and breath awareness or body scanning techniques Control group: - Participants had one month of access to 10 audiobook sessions from the Headspace Guide to Meditation and Mindfulness through the HeadSpace app
Firestone et al. (2018) [28]	cRCT / 1 (after 12 weeks)	New Zealand	*Age:* ≥ 18 years ***Intervention group (n* = *389):*** *Female sex:* 65.8% ***Control group (n* = *405):*** *Female sex:* 65.2% ***Handling of drop-outs:*** No information on drop-outs Per-protocol analysis	- Goal - Reward - Progress - Social sharing	12 weeks	Intervention group: - Participants had 12 weeks of access to the OL@-OR@ m-Health program Comparison group: - Participants received a control version of the OL@-OR@ tool which was similar in visual design but that only collected baseline and outcome data
Flett et al. (2019) [29]^3^	RCT / 2(t_1_ = after 10 days, t_2_ = after 30 days)	New Zealand	*Age:* M = 20.1 (18–49 years) ***Intervention group (n* = *72)*** *Drop-out at t*_*1*_*:* 0% *Drop-out at t*_*2*_*:* 7.0% ***Comparison group (n* = *63)*** *Drop-out at t*_*1*_*:* 0% *Drop-out at t*_*2*_*:* 8.0% ***Control group (n* = *75)*** *Drop-out at t*_*1*_*:* 2.8% *Drop-out at t*_*2*_*:* 10.7% ***Handling of drop-outs:*** Per-protocol analysis	- Reward - Progress - Surprise - Sensation^2^	10 days, up to 40 days	Intervention group (HeadSpace): - Participants needed to complete the introductory level over 10 days. After that, they could continue using the app for 30 more days Comparison group (Smiling Mind): - Participants received the Smiling Mind app with the “For adults” program for 10 min each day over 10 days and could continue using the app for a further 30 days. The program included practices like mindful breathing, body scan, or sitting meditation Control group: - Participants used 40 days the app Evernote while they needed to write down all the things they can remember doing on this day last week for 10 min every day for 10 days
Howells et al. (2016) [30]	RCT / 1 (after 10 days)	11 countries (including Australia, USA, Poland, Switzerland, Malta, Sweden, and Singapore; with no information on further countries)	***Intervention group (n* = *97):*** *Age:* M = 39.7 *Female sex:* 85.6% *Drop-out:* 41.3% ***Control group (n* = *97):*** *Age:* M = 40.9 *Female sex:* 90.7% *Drop-out:* 34.1% ***Handling of drop-outs:*** Per-protocol analysis	- Reward - Progress - Surprise - Sensation^2^	10 days	Intervention group: - Participants needed to follow the daily mindfulness exercises feature of the ‘‘Take 10’’ (introductory level) program for 10 min a day over 10 days Control group: - Participants used the list-making app Catch notes and needed to use the checklist function to ‘create an outline of what they did on this day last week’ for 10 min a day over 10 days
Keeman et al. (2017) [31]^4^	Experimental study with randomization/ 1 (after 1 week)	New Zealand	*Age:* M = 21.5 ***Intervention group (n* = *32):*** *Female sex: 75.0%* ***Control group (n* = *28):*** *Female sex: 71.4%* ***Handling of drop-outs:*** No drop-outs, but removal of 10 participants from analysis (5 for intervention and 5 for control group)	- Levels - Rewards - Leaderboard	1 week	Intervention group: - Participants were required to play the Wellbeing Game every day for seven days. This game contains its own logged activities, primary psychosocial interventions, secondary interventions and different coping strategies Wait-list group: - Participants did not receive any intervention during those 7 days. They just had to complete the survey and an image task at the beginning and after one week (the same as the control condition)
Kelders et al. (2018) [32]^2,5^	RCT / 1 (after 12 weeks)	Nether-lands	***Intervention group (n* = *39)****:* *Age*: M = 23.4 *Female sex:* 71.8% *Drop-out:* 0% ***Control group (n* = *36):*** *Age*: M = 22.2 *Female sex:* 58.3% Drop-out: 2.8% (due to technical issue) ***Handling of drop-outs:*** Per-protocol analysis	- Challenges - Rewards - Progress - Avatar - Storytelling/ Narration - Sensation	12 weeks	Intervention group: - Participants have 12 weeks of access to the self-guided “This is your life” web-based positive psychology intervention, which included 8 lessons with approximately 5 exercises within 2 challenges Comparison group: - Participants received the same intervention but the layout and wording differed from the intervention group so that it was non-gamified
Litvin et al. (2020) [33]	RCT / 2 (after 17 days, after 35 days)	United Kingdom	*Age*: 16 years or older ***Intervention group (n* = *135):*** *Female sex:* 44.4% *Drop-out:* 39.2% ***Control group (n* = *89):*** *Female sex:* 29.2% *Drop-out:* 67.0% ***Wait-list group (n* = *130):*** *Female sex:* 41.5% *Drop-out:* 40.4% ***Handling of drop-outs:*** Per-protocol (complete case) analysis	- Challenges - Rewards - Level - Avatar - Storytelling/ Narration	5 weeks	Intervention group: - Participants used the App “eQuoo” and had to complete 5 levels in 5 weeks. They learned two skills at each level from CBT, positive psychology and systematic therapies Control group: - Participants used the App “CBT Thought Diary”, which is based on CBT and positive psychology. They had to complete a mood diary and do typical CBT exercises Wait-list group: - Participants answered the questionnaires that each group had was required to answer without receiving an intervention
Myers et al (2017) [34]	RCT / 2 (t_1_ = after 30 days, t_2_ = after 60 days)	USA	***Intervention group (n* = *237):*** *Age:* M = 41.58 *Female sex:* 75.1% *Drop-out at t*_*1*_*:* 41.0% *Drop-out at t*_*2*_*:* 46.9% ***Control group (n* = *242):*** *Age:* M = 41.93 *Female sex:* 76.9% *Drop-out at t*_*1*_*:* 32.6% *Drop-out at t*_*2*_*:* 33.5% ***Handling of drop-outs:*** Intention-to-treat analysis + complier analysis	- Challenges - Progress - Social sharing - Sensation	30 days	Intervention group: - Participants had access to 152 challenges designed to increase well-being Control group: - Participants had access to a website with numerous links on the topic of well-being
Przybylko et al. (2021) [35]	RCT / 2 (after 12, after 24 weeks)	Australia	***Intervention group (n* = *255):*** *Age:* M = 49.5 *Female sex:* 69.8% *Drop-out:* 37.6% ***Control group (n* = *253):*** *Age:* M = 45.4 *Female sex:* 73.3% *Drop-out:* 36.0% ***Handling of drop-outs:*** Per-protocol analysis	- Challenges - Rewards - Leaderboard - Social sharing - Sensation	10 weeks	Intervention group: - Participants were given access to “The Live More Project” or “Te Lift Project”. In each weekly session, users viewed 1 out of 10 topic base learning videos and completed daily and weekly challenges Wait-list group: - Participants were placed on a waitlist without access to the intervention
Routledge et al. (2021) [36]	RCT / 1 (after 4 weeks)	Australia	***Intervention group (n* = *170):*** *Age:* M = 42.6 *Female sex:* 66.5% ***Control group (n* = *182):*** *Age:* M = 42.9 *Female sex:* 67.0% ***Handling of drop-outs:*** No detailed information on drop-outs stratified by intervention vs. control available. Therefore, presented sample sizes refer to the samples being analyzed Overall drop-out 53.8% Intention-to treat analysis + Per-protocol analysis	- Progress	4 weeks	Intervention group: - Participants were given access to “MyBrainSolutions” which includes online games addressing cognitive and emotional performance. Users needed to play 2–3 times a week for 20–30 min Wait-list group: - Participants were placed on a waitlist without access to the intervention
Schakel et al. (2020) [37]	RCT / 2 (after 6–7 weeks, after 10 weeks)	Nether-lands	***Intervention group (n* = *35):*** *Age:* M = 22.5 *Female sex:* 0% *Drop-out:* 17.2% ***Control group (n* = *34):*** *Age:* M = 22.9 *Female sex:* 0% *Drop-out:* 8.9% ***Handling of drop-outs:*** Per-protocol analysis	- Goals - Storytelling/ Narration	6 weeks	Intervention group: - Participants received a guided ICBT intervention for 6 weeks, which contained 6 modules guided by a therapist. Additionally, users played a serious game (Ivanovna©) that included comparable modules as the guided intervention Control group: - Participants did not receive any intervention

*RCT* Randomized control trial, *cRCT* Cluster randomized control trial, *M* Median, *f* Female, *m* Male, *CBT* Cognitive behavioural therapy, *ICBT* Internet-based cognitive behavioural therapy

^1^Numbers of participants refer to the time of randomization

^2^Information taken from [38, 39]

^3^In this case only the HeadSpace group is identified as the intervention group because of gamified elements

^4^Only results from the real-life experiment are reported because Study 2 has no control group

^5^This study reports results from a pilot experiment and real-life experiment. We only present results for the real-life experiment here

### Characteristics of primary studies

Overall, four studies originated from the United Kingdom (UK), three each from Australia and New Zealand, two from the Netherlands, one from Portugal and one from the United States of America (USA). One study included participants from eleven countries; for another study, the location could not be determined. In most studies, generally healthy working-aged adults were observed (*n* = 13), while some focused specifically on university students (*n* = 3). Overall, 3,585 participants were included in the studies, with higher percentage of women (62%). The duration of the interventions ranged from 10 min (one break) up to 12 weeks, with most studies lasting four to six weeks (*n* = 8). Moreover, the majority of the studies provided an active control condition (*n* = 12). Of those active control conditions, five studies included access to meditation guides, the provision of information or the filling out of diaries. Further, other active interventions, such as similar applications to the intervention group or apps based on cognitive behavioural therapy (CBT), were visible in four studies. Other comparison groups framing the same intervention just the other way around, or the same intervention with another design or layout (*n* = 3). Seven studies had a waitlist or inactive control condition (without any intervention). While most studies were two-armed, four studies used a three-arm design. All studies identified used randomization for allocation to the groups). A comprehensive overview of the interventions, including their used psychological techniques or strategies, can be found in the supplementary material.

### Outcomes investigated within primary studies

A broad variety of outcomes investigated within the selected studies was observed. Beyond measuring various dimensions of well-being (*n* = 7), the studies also analysed resilience (*n* = 4) and mindfulness (*n* = 1). In addition, stress (*n* = 6), depression (*n* = 5) or anxiety (*n* = 5), as well as other mental health outcomes (*n* = 11) such as satisfaction with life, quality of life, or positive and negative affect were examined within the included studies. Due to the large number of different outcomes, the measurement instruments used were highly heterogeneous. Even for the same outcome, various instruments were used (Table 3). A detailed overview of the outcomes examined can be found in Table 3, while all included outcomes within the studies are presented in the supplementary material.

**Table 3:** Outcomes and scales used within primary studies

Outcome		Scale used	Study	Rating
**Primary**	**Secondary**			
**Resilience/Mindfulness**				
**Resilience**		The Wagnild Resilience Scale (WRS)	Champion et al. (2018) [23]	+
		6-item Brief Resilience Scale	Flett et al. (2019) [29]	o
		12-item Scale of the Resilience Research Centre – Adult Resilience Measure (RRC-ARM), Section C	Litvin et al. (2020) [33]	+
	**Resilience**	Connor–Davidson Resilience Scale (CD-RISC10)	Deady et al. (2022) [26]	+
**Mindfulness**		12-item Cognitive Affective Mindfulness Scale-Revised	Flett et al. (2019) [29]	o
**Well-being**				
**Well-being (subjective, overall, combined)**		SF36v2 short form	Costa et al. (2018) [25]	+
		Individual variables	Firestone et al. (2018) [28]	+
		21 items I COPPE Scale	Myers et al. (2017) [34]	o
		The Short Warwick-Edinburgh Mental Wellbeing Scale (SWEMWBS)	Keemann et al. (2017) [31]	+
	**(Self-reported) Well-being**	WHO Wellbeing Index (WHO-5)	Deady et al. (2022) [26]	+
		Numeric Rating Scale (NRS) on wellbeing	Schakel et al. (2020) [37]	+
**Psychological/Mental well-being**		Individual variables	Firestone et al. (2018) [28]	+
		21 items I COPPE Scale	Meyers et al. (2017) [34]	+
		Warwick Edinburgh Mental Well-being Scale	Bostock et al. (2019) [15]	+
		COMPAS-W	Routledge et al. (2021) [36]	+
	**Psychological/Mental well-being**	Ryff’s Scales of Psychological Well-Being (RPRS)	Litvin et al. (2020) [33]	+
**Spiritual well-being**		Individual variables	Firestone et al. (2018) [28]	+
**Interpersonal well-being**		21 items I COPPE Scale	Myers et al. (2017) [34]	+
**Community well-being**		21 items I COPPE Scale	Myers et al. (2017) [34]	+
		Individual variables	Firestone et al. (2018) [28]	+
**Occupational well-being**		21 items I COPPE Scale	Myers et al. (2017) [34]	o
**Physical well-being**		21 items I COPPE Scale	Myers et al. (2017) [34]	o
		Individual variables	Firestone et al. (2018) [28]	+
**Economic well-being**		21 items I COPPE Scale	Myers et al. (2017) [34]	+
**Stress, anxiety, and depression**				
**Stress**		Stress Overload Scale (SOS)	Economides et al. (2018) [27]	o
		The Perceived Stress Scale (PSS)	Champion et al. (2018) [23]	+
			Flett et al. (2019) [29]	o
		Self-perceived stress	Keemann et al. (2017) [31]	o
		21-item Depression, Anxiety and Stress Scale (DASS-21)	Przybylko et al. (2021) [35]	+
**Psychological distress**		Subscales of the Hospital Anxiety and Depression Scale	Bostock et al. (2019) [15]	+
**Depression**		PHQ-9	Deady et al. (2022) [26]	+
		20-item Center for Epidemiological Studies Depression Scale (CES-D)	Flett et al. (2019) [29]	+
			Howells et al. (2016) [30]	+
		21-item Depression, Anxiety and Stress Scale (DASS-21)	Przybylko et al. (2021) [35]	+
		Depression, Anxiety and Stress Scale (DASS-42)	Routledge et al. (2021) [36]	+
**Anxiety**		Hospital Anxiety and Depression Scale–Anxiety Subscale (HADS-A)	Flett et al. (2019) [29]	o
		21-item Depression, Anxiety and Stress Scale (DASS-21)	Przybylko et al. (2021) [35]	+
		Depression, Anxiety and Stress Scale (DASS-42)	Routledge et al. (2021) [36]	+
	**Anxiety**	Generalized Anxiety Disorder scale (GAD-2)	Deady et al. (2022) [26]	o
		One-item anxiety scale	Litvin et al. (2020) [33]	+
**Other mental health outcomes**				
**Flourishing**^**1**^		8-Item Flourishing Scale	Flett et al. (2019) [29]	o
		Flourishing Scale	Howells et al. (2016) [30]	o
**Satisfaction with life**		Satisfaction with Life Scale (SWLS)	Champion et al. (2018) [23]	+
			Howells et al. (2016) [30]	o
			Przybylko et al. (2021) [35]	+
**Quality of life**		WHOQOL-BREF is a 26-item scale	Costa et al. (2018) [25]	+
		Short Form Health Index (SF-36)^2^	Przybylko et al. (2021) [35]	+
	**Quality of life**	RAND-36	Schakel et al. (2020) [37]	o
**Energetic arousal**		Activation-Deactivation Adjective Checklist (ADACL)	Collins et al. (2019) [24]	+
**Recovery**		Recovery experience scale	Collins et al. (2019) [24]	+
**Positive and negative affect**		Scale of Positive and Negative Experience (SPANE)	Economides et al. (2018) [27]	+
		Positive and negative affect (PANAS)	Kelders et al. (2018) [32]	+
		Positive and Negative Affect Schedule^1^	Schakel et al. (2020) [37]	o
**Negative affect**		Positive and negative affect (PANAS)	Howells et al. (2016) [30]	o
**Positive affect**		Positive and negative affect (PANAS)	Howells et al. (2016) [30]	+
**Cognitive engagement**		Short version of the Personal Involvement Inventory (PII)	Kelders et al. (2018) [32]	+
**Cognitive and affective engagement**		Flow State Questionnaire of the Positive Psychology Lab (PPL-FSQ)	Kelders et al. (2018) [32]	+
**Frustration and irritability**		Brief Irritability Test (BITe)	Economides et al. (2018) [27]	+
**Emotional cognition**		WebNeuro assessment tasks	Routledge et al. (2021) [36]	+
	**Personal growth**	Personal Growth Initiative Scale (PGIS)	Litvin et al. (2020) [33]	+
	**Sleep problems**	Medical Outcomes Study Sleep Scale	Schakel et al. (2020) [37]	+

** + **Significant positive changes in at least one of the measurements of the relevant outcome

o No significant changes in the relevant outcome

^1^Used as a proxy for well-being, among other things

^2^Used as proxy for mental health

### Effectiveness of gamified digital interventions

#### Resilience and mindfulness

In total, four studies included resilience and one study mindfulness. While significant effects were found with respect to resilience for most studies, one of the four studies measuring resilience did not show significant improvements [29]. Moreover, the study by Flett et al. [29] also failed to identify any significant effects on mindfulness.

### Well-being

Overall, seven studies investigated well-being within their studies. Most of these studies showed significant positive effects through game-based interventions. The general well-being significantly improved in five studies [25, 26, 28, 31, 37], while all studies examining psychological or mental well-being (*n* = 5) found significant improvements [15, 28, 33, 34, 36]. Spiritual well-being (*n* = 1) [28], interpersonal well-being (*n* = 1) [34], community well-being (*n* = 2) [28, 34], and economic well-being (*n* = 1) [34] showed also significant progresses through a game-based intervention. In contrast, for occupational well-being no significant effects could be observed [34]. Physical well-being was significantly improved in one study [28], while no effects could be observed in another [34].

### Stress, depression, and anxiety

In total, six studies examined stress and five studies each anxiety and depression. There is some evidence suggesting that game-based interventions have significant positive effects on (psychological di-)stress (*n* = 3) [15, 23, 35]. With regard to the internalizing mental health problems, positive effects on depression (*n* = 5) [26, 29, 30, 35, 36], and anxiety (*n* = 3) [33, 35, 36] were observed. Results related to stress were very mixed, with three studies showing significant improvements [15, 23, 35] and three other studies without significant effects [27, 29, 31]. All studies used different scales. The results regarding anxiety are similar: Three studies reported significant positive effects, while two studies could not identify any significant improvements. In contrast, all interventions had a significant positive impact on the prevention of depression regardless of the measurements used.

### Other mental health outcomes

Next to the mental health outcomes reported, a diverse array of emotional outcomes could be identified within eleven studies. Flourishing partly used as a proxy for well-being, was investigated in two studies. However, no significant results were identified [29, 30]. Three studies examined satisfaction with life and one study quality of life, while findings are heterogeneous. Using the same satisfaction with life scale, two studies detected significant improvements [23, 35], while one study did not [30]. Meanwhile, quality of life was significantly improved within two gamified interventions [25, 35]. However, one study did not show any significant improvements [37]. Personal growth (= 1) [33], sleep problems (*n* = 1) [37], emotional cognition (*n* = 1) [36], frustration and irritability (*n* = 1) [27], cognitive engagement (*n* = 1) [32], cognitive and affective engagement (*n* = 1) [32], energetic arousal (*n* = 1) [24], and recovery (*n* = 1) [24], were identified in one study each and had a significant association with gamified interventions. At the same time, positive and negative affect, sometimes used as proxy for well-being, were included in three studies as a combination and in one study separately. Accordingly, there was a significant association for positive and negative affect visible in two studies [30, 32]. Interestingly, Howells et al. [30] reported significant improvements for positive affect but not negative affect. In turn, Schakel et al. [37] could not identify any significant effects, neither for negative nor for positive affect (Table 3).

### Game elements within primary studies

**Fig. 2:**
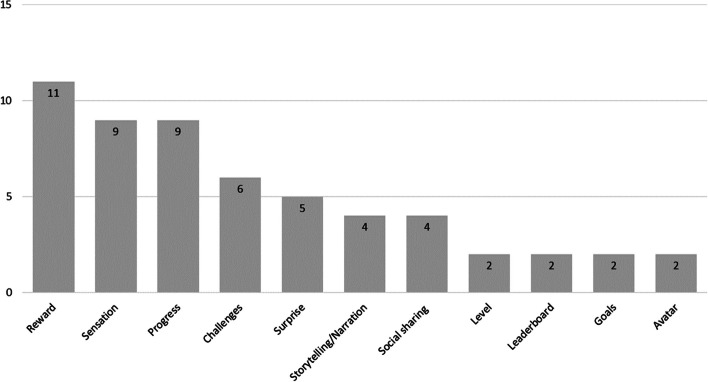
Game elements observed in the studies

In total, eleven game elements were applied within the studies included in this review. The most frequently utilized elements were reward (*n* = 11), progress and sensation (*n* = 9), followed by challenges (*n* = 6), surprise (*n* = 5) social sharing and storytelling/narration (n = 4). Less frequently used were avatars, goals, leaderboards and levels (*n* = 2) (Fig. 2). Overall, at least three game elements were integrated in the interventions, while Kelders et al. [32] used most game elements (*n* = 6).

Regarding the use of gamification, an analysis of the elements shows great variations. For example, Routledge et al. [36] showed with the integration of progress a significant improvement in psychological well-being. At the same time, Myers et al. [34] demonstrated that an integration of four elements (Challenges, Progress, Social sharing, and Sensation) also improve psychological well-being. Moreover, when integrating challenges exclusively, significant improvements for well-being and depression were observed but not for anxiety [26]. Costa et al. [25] pointed out, that the integration of four elements (Challenges, Storytelling, Social sharing, and Sensation) similarly improve well-being.

### Quality appraisal of included studies

**Fig. 3:**
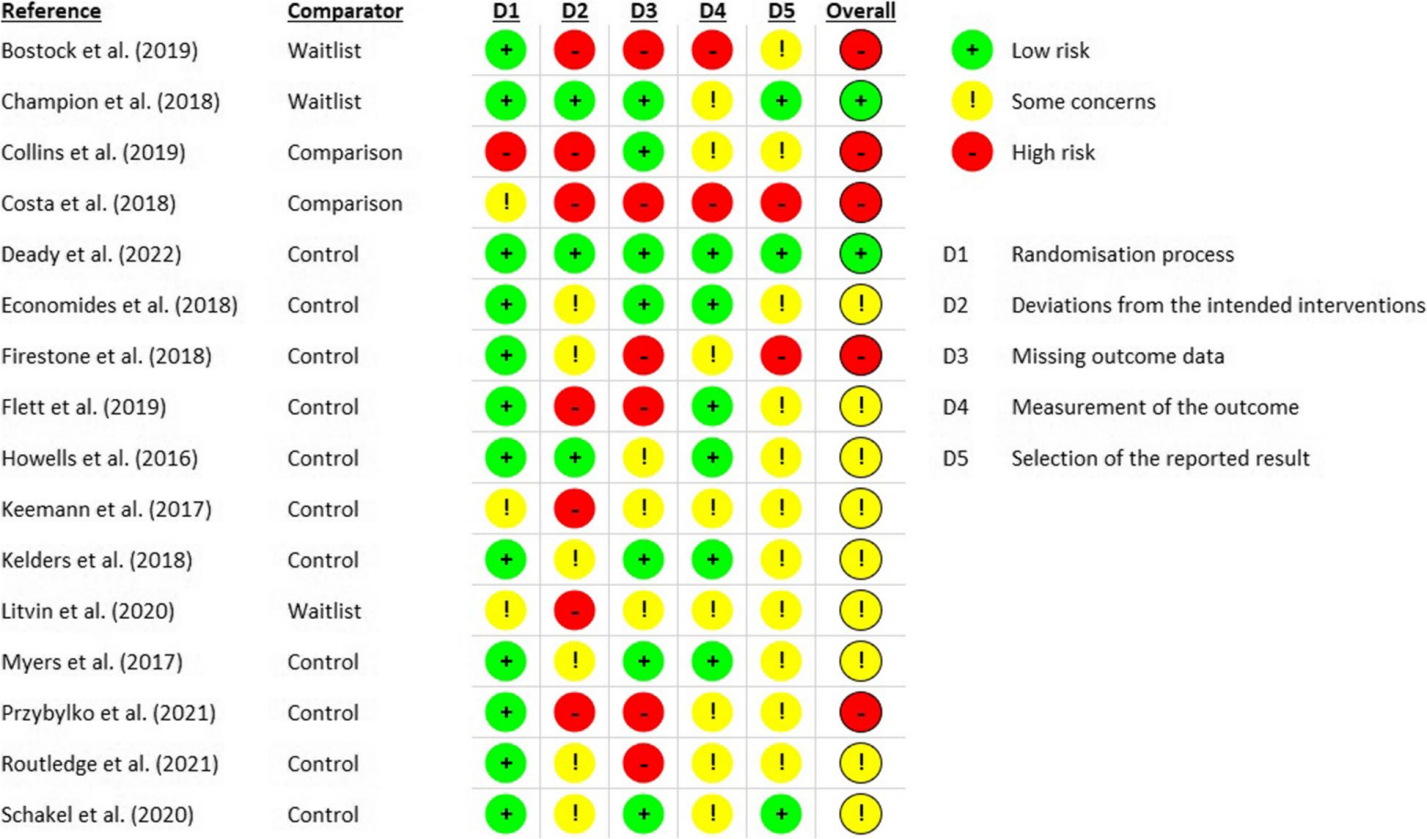
Quality appraisal of includes studies

Overall, the studies showed some or high risk of bias, particularly due to deviations from the intended interventions or missing outcome data. In contrast, the randomization only led to low risk of bias in most of the studies. There was a study which showed only low risk for all five dimensions, whereas one study showed high risk in four dimensions and some concerns in terms of the randomization process (Fig. 3).

In addition to this quality assessment, we have extracted the drop-out rates. Drop-outs varied from 0 to 67.0% per study. Four studies did not report on drop-outs, and two studies attributed the drop-out explicitly to technical issues. Most studies performed per-protocol analysis or are based on complete data. Only three studies conducted both an intention-to-treat and per-protocol analysis; one further study performed an intention-to-treat analysis merely.

## Discussion

This scoping review investigated the effects of gamified digital interventions in mental health promotion and prevention of mental diseases among working-age adults. Further, it investigated which game elements were most commonly used. Overall, positive effects of gamified interventions on mental health were identified, in particular on psychological well-being and depressive symptoms. However, further outcomes indicated heterogenous findings. Most game elements used were reward, sensation, and progress. However, due to missing information in the primary studies, no substantiated conclusion about the (optimal) quantity or composition of game elements in an intervention can be drawn.

According to our research, this is the first scoping review which investigates the effectiveness of gamified digital interventions on mental health in adults of working age from a health promotion and disease prevention perspective. Previous reviews which, however, did not concentrate on health promotion and disease prevention, underline our findings in a very general way [11, 12, 40, 41]. However, when interpreting the results in the context of mental health, one needs to take into account that we explicitly focussed on persons who showed no mental impairments and, therefore, people who have high scores on a well-being scale and low scores on a depression scale. These floor or ceiling effects may – in contrary to treatment in mentally ill persons – only lead to a limited room for improvement. For that reason, one should not merely focus on statistical significance, but also on effect sizes. However, also small effect sizes may be considered as relevant.

When interpreting the overall results, several further aspects need to be considered. First, the intervention period is relatively short in all included studies. Only two studies had an intervention period of 12 weeks [28, 32]. Five studies of four weeks, or 30 days respectively [23, 26, 27, 34, 36], and four studies of only ten days or less [24, 29–31]. There are indications that interventions with longer duration are more effective than those of shorter length [[31], e.g. [26, 30, 34]]; e.g. interventions with a duration of more than one month did not show non-significant results in mental health-related outcomes [e.g. [15, 25, 33]]. Moreover, taking into consideration that half of the studies did not have an active control group [15, 23, 31, 35–37], the findings need to be interpreted with caution. The inactive control group can be compared with the intervention group, however, in these cases no assertion can be made if the gamified intervention is more effective compared to a non-gamified intervention.

Although it has to be acknowledged that all intervention studies included in the synthesis used randomization for allocation purposes, high dropout rates in the majority of the studies should be kept in mind. Drop-out rates might be higher in health promotion and disease prevention than in treatment, due to low psychological strain and lower motivation [42]. However, only four studies conducted an intention-to-treat analysis to avoid systematic error caused by drop-outs.

Mental health is influenced by numerous risk and protective factors that interact with each other [43]. In this respect, different determinants, such as social conditions, working or living conditions, could influence the effectiveness of game elements in terms of well-being. Myers et al. [34], for instance, found a statistically significant relation with income as well as community and economic well-being. Thus, high-income earners were 2.34 times more likely than low-income earners to comply with the programme. These effects – as already described by Dahlgren and Whitehead [44] – was taken up in a model on digital determinants of health equities [45]. Beyond that, it might be interesting to examine a person’s individual characteristics in the context of gamification. It is thereby possible that e.g., personality, level of knowledge, experiences or even level of motivation, may influence the effectiveness of an intervention. As an example, interventions could be more effective for individuals who already enjoy playing games in their free time [46]. Since too little information on other variables was provided, no conclusive statement regarding these indicators can be drawn.

As a matter of fact, a (long-term) impact of an intervention is one of the most important aspects. The longest follow-up in the included studies was 12 months [26]. Other longest follow-up periods were 12 weeks [35], 60 days [34], 30 days [29], and four weeks [37]. However, most studies do not report follow-up measurements. For this reason, none of the studies investigated whether well-being is increased in the long term or whether intrinsic motivation is maintained. This aspect is a key element in the health care sector. Plugmann [16] emphasizes that gamification can help to reduce costs in the field of prevention. The authors argue that new products and services with gamification can lead to a breakthrough as an innovative prevention strategy. Thereby, however, it is a prerequisite that individuals are willing to share their data. A survey examining the usage of big data and in relation the protection of the privacy indicated that two third of respondents believed that too little attention is paid to the enforcement of data protection and that it will therefore fail. On a positive note, however, the healthcare industry has the highest level of trust compared to other industries, at over 20% [47]. None of the studies included investigates the aspect of cost savings and trusts in digital interventions. In this respect, a long-term view of cost savings and the presentation of the tolerance level when opening private data is an important aspect that could be decisive for the success of gamified interventions in mental health.

### Limitations

There are some limitations which have to be taken into account when interpreting the results. First, the included sample covers a wide age range. We focused on people in the working age group (18–65), whereas young individuals have different habits and needs compared to, for example, individuals nearing the age of retirement. Accordingly, a differentiation of age would be necessary in order to address the effectiveness more specifically. Along these lines, the whole age range was not included in any of these studies. Participants in the study of Costa et al. [25] for example, had a mean age of 73 years, which is attributable to their inclusion criteria (study population should be 50 years or older). Thus, participants above the maximum age of 65 years were included. In addition, some studies focused on university students, with a mean age of e.g. 21.48 years in Keeman et al. [31] and 22.8 years in Kelders et al. [32]. So, within this review, no differentiation of age groups was done. It is therefore critical to consider whether the sample was defined too broadly or whether a differentiated presentation is necessary.

Second, about half of the studies comprised small sample sizes of fewer than 100 participants [23–25, 27, 31, 32, 37]. However, some studies investigated more than 300 participants [33–36], whereas Firestone et al. [28] counted actually 794 participants. The heterogeneous number of included participants was not taken into account and thus no weighting of the results was performed. Due to this and the heterogenic target sample, the generalisation of findings is limited.

Third, a limitation arise from the lack of comparability of the studies, as different survey and evaluation instruments were used. For instance, the overall well-being counts four different scales for four measurements. Similar observations were made for stress, depression, and anxiety.

Fourth, all studies reported at least one significant result. This might be an indication of publication bias.

Furthermore, a variety of statistical analyses were used. This results in a fifth and major limitation. Ratings were presented as a plus (significant positive effect), if one out of various analysis found a significant impact. Since the analysis were very heterogeneous, no differentiation between the analysis and the number of significant results has been made. Therefore, the conclusion about the strength of the effects is limited.

Sixth, several studies used HeadSpace as a gamified intervention [15, 23, 24, 27, 29, 31]. Thus, due to the high number of studies based on the HeadSpace, the variety of gamified interventions is limited, resulting in a potential for bias as this intervention and its effects are given a higher weighting.

Seventh, the usage of game-based elements is diverse and no constant findings were observed. In some cases, especially concerning HeadSpace, the elements used are not described clearly, and different wordings are used. Thus, findings addressing these elements are insufficient, which indicates the need to a well-developed categorization or taxonomy for game elements to make clear statements about the number, type, and combinations of elements which are effective for promoting mental health and preventing mental diseases.

Finally, the quality assessment revealed some or high risk of bias, which must be taken into account when interpreting the results. Although we decided to not only focus on randomized controlled trials, but to include all studies with a control condition, all studies used randomization for allocation. The study quality is heterogeneous, but not that bad as one might expect from experiences of digital interventions in the previous decades. Therefore, as more studies become available, a more detailed perspective on study design and statistical analysis should be considered.

## Conclusions

There is evidence of the effectiveness of digital game elements in improving mental health among working-age adults. However, findings are still limited, and results are heterogeneous, which can be traced back to the different interventions and designs applied within the included studies. A variety of eleven elements was used, and it was not clear whether some features or combinations are more important in the context of mental health promotion and prevention.

Despite the limited research field, the present review indicates important insights and tendencies for research and practice. Thus, especially sociodemographic variables, such as a differentiation between age groups, should be considered in future research. In the course of the next few years, it will be important to identify the long-term effects in order to expand the innovation capability described above. Accordingly, this means for policy to support digital gamified interventions, in research and in practice, to promote one’s mental health. The implementation of digital interventions for treatment presents one important step in the right direction. Now, gamification should become more of a focus. Another important aspect that was taken up is the satisfaction of basic needs. In future work, the reference to self-determination theory could be more strongly focussed, whereby a more well-founded statement could be made with regard to the number and combination of game elements. Along these lines, a taxonomy is needed to adequately address relevant game design elements in the field of mental health promotion and prevention.

For the practice of health promotion and prevention, the increasing digital innovations result in new interfaces that need to be linked in the future. For instance, data protection is more important than ever, and the success of gamified interventions is related to the trust of users. Among other things, the current information overload is an important issue. Therefore, high-quality interventions need to be made transparent. With regard to reaching specific groups of people and associated effects, prevention and health promotion also face innovative strategies. In conclusion, some gaps with considerable potential for further research and practice in health promotion and prevention can be identified in this still very young field of research.

### Supplementary Information

**Additional file 1. **

## Data Availability

All data is described in the manuscript and the supplementary material.
