# Supplementary material for: Effectiveness of gamified digital interventions in mental health prevention and health promotion among adults: a scoping review
Source: BMC Public Health. 2024 Jan 2;24:69. doi: 10.1186/s12889-023-17517-3 (PMC10763397; doi:10.1186/s12889-023-17517-3)
Supplement: Supplementary file 1 — Additional file 1. [file 12889_2023_17517_MOESM1_ESM.pdf]

Description of used game elements

Reward[1]	Also known as badges, medals, trophies and achievements. It is a kind of extrinsic feedback that praises the players' specific set of actions, e.g. completing a certain number of problems may lead them to earn a "Solver" badge; finishing a task in a predefined time limit may earn them a "Flash" trophy (Todas et al. 2019)
Sensation	This is either visual or sound stimulation, etc. It is related to the use of learners' senses to improve the experience (intrinsic). This can be done through dynamic and gameful interfaces, Virtual Reality (VR) and/or Augmented Reality (AR) (Toda et al. 2019)
Progress[2]	Milestone that determines progressive stages in the game/context (Hervas et al. 2017); Also known as progress bars, steps, maps. Provides an extrinsic guidance to the users of their advance in the environment, allowing these users to locate themselves (Todas et al. 2019)
Challenges	Journey of actions that a user must overcome (Hervas et al. 2017)
Surprise	Parts of the context that are not expected by the users, like special rewards or hidden elements (Hervas et al. 2017)
Storytelling/Narration	Defines the story, context and all its components (Hervas et al.); Also known as karma system, implicit decisions, etc. This intrinsic concept is the order of events as they happen in the game, through the user experience. This experience is influenced by implicit choices made by the user (Narrative); Can be seen as audio queues, text stories, etc. It is the way the story of the environment is told (as a script). It is told through text, voice, or sensorial resources (Storytelling) (Todas et al. 2019).
Social sharing	Mechanics to share the progress or status externally (Hervas et al. 2017)
Level	Subdivision of complex elements in simpler ones to control progress, learning and difficulty (Hervas et al. 2017); Also known as skill level, character level etc. This is related to an extrinsic hierarchical layer that provides the user new advantages as they advance in the environment (Todas et al. 2019)
Leaderboard	Way to show the leading competitors (Hervas et al. 2017)
Goals[3]	Can be seen as audio queues, text stories, etc. It is the way the story of the environment is told (as a script). It is told through text, voice, or sensorial resources (Todas et al. 2019)
Avatar[4]	The assumed or expected functions played by a user in a specific situation (Hervas et al. 2017)
[1]In Hervas et al. 2017 known as Achievements<br>[2]In Todas et al. 2019 known as Acknowledgement<br>[3]In Todas et al. 2019 known as Objectives<br>[4]In Hervas et al. 2017 known as Role	

Description of interventions

Study	Game-based elements	Description of game elements and further features	
Bostock et al. 2019	Reward<br>Progress<br>Surprise<br>Sensation	HeadSpace: Mindfulness Meditation App<br>- Completion of meditation sessions (by choosing own topic of interest)<br>- Targets and progress are shown clearly<br>- Live display with all active users meditating can be seen and sessions be joined<br>- Software tracks users' meditation sessions for motivation to perform them daily and provides rewards for meditation streaks<br>- New unpredictable meditations to explore<br>- Educational videos and animations, colourful aesthetic, minimal text	Follows a guided mindfulness meditation (MM) delivered by former Buddhist monk Andy Puddicombe with an introduction of the key principles of mindfulness by using a two-component model of mindfulness.
Champion et al. 2018	Reward<br>Progress<br>Surprise<br>Sensation	HeadSpace: Mindfulness Meditation App<br>- cf. Bostock et al. 2019	Cf. Bostock et al. 2019
Collins et al. 2019	Reward<br>Progress<br>Surprise<br>Sensation	HeadSpace: Mindfulness Meditation App<br>- cf. Bostock et al. 2019	Cf. Bostock et al. 2019
Costa et al. 2018	Challenges<br>Storytelling<br>Social sharing<br>Sensation	Game-based Learning Platform (GBLP):<br>- Set of missions related to physical and cognitive activity<br>- Mini-games to train sensation, perception, attention and memory<br>- Storytelling: Time-travelling experience to Hizen 1709 or London 1895 (with a non-player character),<br>- Social sharing: Users can check their progress and daily-life missions and post a message on the web platform	Follows the dimensions of health, security and participation in society.

		Computer-assisted Platform (CAP)<br>- Videos concerning nutrition, human security and related to the benefits of physical activity, biological effects of the ageing process and fall prevention<br>- Storytelling: Paris 1948<br>- Mini-games and quiz<br>- Social sharing: Users can share their progress, manage learning events, share information and their doubts about the different modules in the CAP	
Deady et al. 2022	Challenges	HeadGear: Mobile-based intervention focusing on mindfulness and behavioral activity<br>- Challenges (evidence-based therapeutic techniques) have to be completed for 5-10 min every day<br>- Risk calculator for mental disorders and personal feedback<br>- Mood tracker and toolbox of skills	Centered on behavioural activation and mindfulness.
Economides et al. 2018	Reward<br>Progress<br>Surprise<br>Sensation	HeadSpace: Mindfulness Meditation App<br>- cf. Bostock et al. 2019	Cf. Bostock et al. 2019
Firestone et al. 2018	Goals<br>Reward<br>Progress<br>Social sharing	OL@-OR@ app: M-health program<br>- Provided information on healthy eating and physical activity, culturally relevant information, and links to local activities and services<br>- Supports users to set goals/challenges for changing health behaviours and identifying steps needed to reach their goals<br>- Participants were encouraged to invite others<br>- Lifestyle trackers for monitoring progress<br>- Tailored tips on mentioned topics<br>- Regular motivational messages<br>- Virtual rewards when goals were achieved<br>- Content can be shared with community group<br>(Ni Mhurchu et al., 2019)	Focuses on managing or reducing the key risk factors for NCDs (eg, diet, physical activity, smoking, alcohol). The Co-design follows the Pacific model of health, including four dimensions of health, namely: spiritual, physical, mental and other.

Flett et al. 2019	Reward<br>Progress<br>Surprise<br>Sensation	Headspace: Mindfulness Meditation App<br>- cf. Bostock et al. 2019	Cf. Bostock et al. 2019
Howells et al. 2016	Reward<br>Progress<br>Surprise<br>Sensation	HeadSpace: Mindfulness Meditation App<br>- cf. Bostock et al. 2019	Cf. Bostock et al. 2019
Keeman et al. 2017	Level<br>Rewards<br>Leaderboard	The Wellbeing Game: Online game, using the Five Ways to well-being<br>- Logging participated activities<br>- Developing relationships within teams that promote good functioning and a good social climate<br>- Promoting mindfulness and building positive emotions<br>- Teaches techniques to address symptoms of strain<br>- Point system for the duration of the recorded activities<br>- Leaderboard<br>- Rewards in form of badges when specific thresholds are achieved	Follows the Five Ways of Well-being (Connect, Be Active, Take Notice, Keep Learning, and Give) to promote mental well-being.
Kelders et al. 2018[1]	Challenges<br>Rewards<br>Progress<br>Avatar<br>Storytelling/Narration<br>Sensation	This is your life: Web-based mental health intervention<br>- Storyline is a user on a journey toward a flourishing life (visualized as a map) and travelling to various destinations (lessons)<br>- Guided by an Avatar "Professor Happiness"<br>- Own participant avatar can be created (but not in experiment)<br>- Introduction and 8 lessons (with approx. 5 exercises and 2 key challenges) that could be completed in 12 weeks<br>- Tailored feedback about how to best perform exercises at various points during each lesson<br>- Badges for completion of lessons<br>- Progress bar and key for finishing mandatory activities	Follows the strategy of Positive Psychology to improve well-being.

Litvin et al. 2020	Challenges<br>Rewards<br>Level<br>Avatar<br>Storytelling/Narration	eQuoo: Mobile mental health well-being app<br>- 5 levels with each two psychological skills extracted from CBT therapies, positive psychology therapies, and systemic therapies<br>need to be done over 5 weeks<br>- Avatar (Dr. Joy): introduces and teaches the skills while using cartoon stick figures<br>- The player's knowledge is tested by two other characters<br>- After the competition of skills a challenge has to be completed (choose-your-own-adventure) by answering questions<br>- Points as coins<br>- Badges ranging from "beginner" to "self-aware".<br>- Varying narratives for each level<br>- Personalization as generalized feedback of their Big Five personality type<br>- Customization in allowing users to choose their own mini-avatar<br>- Mini-games where players can deepen skillsets	Uses two psychological skills extracted from CBT therapies, positive psychology therapies, and systemic therapies.
Myers et al. 2017	Challenges<br>Progress<br>Social sharing<br>Sensation	Fun For Wellness (FFW): Online intervention program<br>- 152 challenges to promote multidimensional well-being<br>- This includes watching vignettes performed by professional actors, watching and/or reading mini-lectures narrated by a coach, engaging in self-reflection exercises and chat rooms, playing interactive games<br>- Participants were not told how many challenges to complete and self-selected which post-introductory challenges to complete<br>- Completion of challenges was tracked using a scoring system	Follows the multiple dimensions of subjective well-being purportedly measured by the I COPPE Scale. Further, the acronym BET I CAN was developed, which stands for Behaviors, Emotions, Thoughts, Interactions, Context, Awareness, and Next Steps.

Przybylko et al. 2021	Challenges<br>Rewards<br>Leaderboard<br>Social sharing<br>Sensation	The Live More Project: Online interdisciplinary intervention<br>- The program integrates strategies from Lifestyle Medicine and Positive Psychology and uses an experiential pedagogical framework of Learn, Experience, Tink, and Share (LETS) facilitated through an e-learning management system<br>- Each weekly session included an educational video of mental health and emotional wellness promotional strategies and completing daily and weekly challenges<br>- For successful completion participants were awarded with challenge points presented on a leaderboard<br>- Social forum for interaction<br>- Reading material, e-workbook for journaling and reminder notifications	Uses strategies from the Lifestyle Medicine and Positive Psychology.
Routledge et al. 2021	Progress	MyBrainSolutions: Online brain training<br>- Participants can choose from several brain training games and activities targeting emotion, feeling, self-regulation<br>- Tracking improvement via WebNeuro (Gordon et al., 2013)	Follows the domains of emotion, thinking, feeling and selfregulation.
Schakel et al. 2020	Goals<br>Storytelling/Narration<br>Serious Game	ICBT and Ivanovna©: Guided internet intervention and Serious Game<br>- 6 modules (goal setting, healthy food and exercise, relaxation, sleep, cognition and worldview, and long-term goals)<br>- Guided by a therapist from whom participants received homework assignments and asynchronously provided feedback messages<br>- Serious game (ViaNova©) was played additionally	Follows the strategy of a cognitive behavioural therapy (CBT) via the internet (ICBT).
